# A double-blind, placebo-controlled study evaluating the effects of caffeine and L-theanine both alone and in combination on cerebral blood flow, cognition and mood

**DOI:** 10.1007/s00213-015-3895-0

**Published:** 2015-03-13

**Authors:** F. L. Dodd, D. O. Kennedy, L. M. Riby, C. F. Haskell-Ramsay

**Affiliations:** Brain, Performance and Nutrition Research Centre, Faculty of Health and Life Sciences, Northumbria University, Newcastle upon Tyne, NE1 8ST UK

**Keywords:** Caffeine, L-theanine, Cognition, Mood, NIRS, Cerebral blood flow, Consumers, Non-consumers

## Abstract

**Rationale:**

Evidence suggests interactive effects of the tea components caffeine and L-theanine on behaviour, yet no data exists exploring the impact of the two on cerebral blood flow (CBF).

**Objectives:**

The current placebo-controlled, double-blind, counterbalanced, crossover study examined the effects of caffeine and L-theanine on CBF and extended previous cognitive and mood findings by using lower doses than previous studies of a similar methodology, which more closely reflect the ratios present in tea.

**Methods:**

Twelve habitual consumers and 12 non-habitual consumers of caffeine each received 75 mg caffeine, 50 mg L-theanine, 75 mg caffeine plus 50 mg L-theanine, and placebo in a counterbalanced order across four separate visits. CBF was measured via near-infrared spectroscopy with cognition and mood assessed at baseline and 30 min post-dose. Salivary caffeine and peripheral haemodynamics were co-monitored.

**Results:**

Caffeine reduced oxygenated haemoglobin (oxy-Hb), increased deoxygenated haemoglobin (deoxy-Hb), improved performance on attention tasks and increased overall mood ratings. Increases in deoxy-Hb following caffeine were more pronounced in non-consumers. Some evidence for increased deoxy-Hb remained when caffeine was combined with L-theanine, but this effect was attenuated and the effects of caffeine on oxy-Hb, cognition and mood were eradicated.

**Conclusions:**

Combining L-theanine with caffeine, at levels and ratios equivalent to one to two cups of tea, eliminated the vasoconstrictive effect and behavioural effects of caffeine. This supports previous findings of an interaction between these substances, despite a lack of effects of L-theanine in isolation. However, at the levels tested here, this did not lead to a positive impact on behaviour.

## Introduction

Caffeine is the most widely consumed psychoactive substance in the world, with coffee and tea representing our main dietary sources (Fredholm et al. 1999). The mechanism by which caffeine exerts its effects is now largely accepted to be through non-selective antagonism of adenosine A_1_ and A_2A_ receptors (Fredholm et al. 1999), with A_1_ receptors being more closely related to neural activation and A_2A_ receptor antagonism leading to vascular effects, including vasoconstriction and a reduction in cerebral blood flow (CBF) (Dunwiddie and Masino 2001). Functional MRI (fMRI) studies have identified that both actions can modulate the blood-oxygenation-level dependent (BOLD) signal response and that the ratio of adenosine receptors (A_1_ to A_2A_) influences the overall vascular and neural effects (Chen and Parrish 2009a; Laurienti et al. 2003), with upregulation of A_1_ receptors observed following chronic caffeine use (Johansson et al. 1993; Shi and Daly 1999). Caffeine has been shown to reduce cerebral blood velocity and CBF assessed using a number of different techniques (Chen and Parrish 2009b; Hasse et al. 2005; Kennedy and Haskell 2011; Laurienti et al. 2003; Mathew and Wilson 1991; Rack-Gomer et al. 2009). A linear dose relationship has been observed between reductions in CBF and caffeine following doses of 1, 2.5 and 5 mg/kg caffeine (Chen and Parrish 2009a). Effects of caffeine on CBF are also dependent upon the level of caffeine habitually consumed. In a withdrawn state, habitual high consumers exhibit higher resting CBF as compared to low consumers, and caffeine use is significantly positively correlated with CBF following both placebo and caffeine (Addicott et al. 2009; Field et al. 2003). There is also evidence that, despite higher CBF overall, high consumers exhibit a greater acute reduction in CBF than low consumers in response to a 250 mg dose of caffeine administered whilst in a state of withdrawal (Field et al. 2003). The reduction in CBF following caffeine runs counter to neurovascular coupling, a complex and poorly understood sequence of processes, whereby an increase in neuronal activation leads to an increase in CBF in order to meet, and, in the case of oxygen, exceed, the metabolic demands presented by the activation. This process typically results in an increase in cerebral oxygenated haemoglobin and a corresponding dilution of deoxygenated haemoglobin. Despite the observed reduction in CBF, and therefore decreased supply of metabolic substrates, caffeine has consistently been shown to improve reaction times (Childs and de Wit 2006; Haskell et al. 2008; Smit and Rogers 2000) and alertness (Quinlan et al. 2000; Rogers 2007). However, debate continues as to whether these well-established effects are dependent upon withdrawal (e.g. Heatherley et al. 2005; Rogers et al. 2003) or not (e.g. Christopher et al. 2005; Haskell et al. 2005). Although the beneficial effects of caffeine on cognition and mood have been reported in a number of studies, relatively few studies have looked at the effects of caffeine in combination with other compounds (see Haskell et al. 2013 for review), despite the fact that caffeine is seldom consumed in isolation.

L-theanine is a naturally occurring amino acid found almost uniquely in tea (*Camellia sinensis*), where it co-exists with caffeine. Its chemical structure is similar to that of the neurotransmitter glutamic acid (Nathan et al. 2006), and it has been shown to increase dopamine concentrations in the rat brain in a dose-dependent manner (Yokogoshi et al. 1998), and to protect against human neuronal cell death in vitro (Cho et al. 2008). However, unlike caffeine, research into the effects of L-theanine in humans is limited. Anxiolytic effects have been reported, whereby 200 mg L-theanine reduced acute stress responses (subjective perception, heart rate and salivary immunoglobulin A) induced by a mental arithmetic task (Kimura et al. 2007), and a 250 mg dose of L-theanine slowed reaction time on a visual probe task, indicating reduced anxiety (Rogers et al. 2008). EEG studies have also provided some support for these findings with increases in resting alpha activity observed following 50 mg (Nobre et al. 2008) and 200 mg L-theanine (Juneja et al. 1999), which the authors interpret as being indicative of relaxation. A dose of 250 mg of L-theanine has also been shown to decrease tonic alpha activity during task performance (Gomez-Ramirez et al. 2007, 2009).

In terms of cognitive function, L-theanine in isolation has been shown to engender decrements in performance (Gomez-Ramirez et al. 2007; Haskell et al. 2008) or, at best, an absence of effects (Gomez-Ramirez et al. 2009; Haskell et al. 2008; Kelly et al. 2008; Owen et al. 2008). However, when administered together, L-theanine modulates or potentiates the effects of caffeine. For instance, Haskell et al. (2008) reported that a number of effects were evident following a combination of 250 mg L-theanine and 150 mg caffeine that were not apparent when each treatment was administered alone; these included increased speed on several tasks, improved semantic memory and increased alertness. Other studies have employed lower doses of L-theanine (~100 mg) and caffeine (~50 mg) to explore these effects (Einother et al. 2010; Kelly et al. 2008; Owen et al. 2008), providing support for the findings from studies of higher doses, in terms of improved accuracy (Einother et al. 2010; Kelly et al. 2008; Owen et al. 2008) and speed (Einother et al. 2010; Owen et al. 2008) on tasks of attention, and improvements to measures of memory (Owen et al. 2008). L-theanine has also been shown to antagonise the physiological effects of caffeine. Rogers et al. (2008) demonstrated that although systolic and diastolic blood pressure were significantly higher following the administration of 250 mg caffeine when compared to placebo or 200 mg L-theanine alone, the combination of the two compounds led to an attenuation of this effect.

A previous study using the dose of caffeine employed here (75 mg) showed that caffeine significantly reduced total haemoglobin as measured by near-infrared spectroscopy (NIRS) (Kennedy and Haskell 2011). Given that co-administration of L-theanine with caffeine has previously been demonstrated to attenuate its haemodynamic effects (Rogers et al. 2008), it remains a possibility that co-administration may also attenuate the cerebro-vascular effect of caffeine. The doses of L-theanine/caffeine and the ratios in which they have been administered in the majority of assessments of these two interventions to date have tended to contain higher levels of L-theanine than caffeine. This is not representative of the ratios normally found in tea in our diet, which tend to be in the region of 2 to 1 in favour of caffeine. Similarly, the lowest dose of L-theanine explored previously in terms of behaviour (100 mg) is equivalent to ~4 cups of tea. The current study aimed to address these issues using NIRS to measure CBF in the pre-frontal cortex during cognitive task performance following caffeine and L-theanine, both alone and in combination. The cohort included both habitual tea drinking consumers and non-habitual consumers of caffeine and involved administration of doses and ratios of caffeine and L-theanine that more closely reflect those present in tea. It is predicted that combining caffeine with L-theanine will enhance effects of caffeine on behaviour whilst diminishing haemodynamic effects. Effects on behaviour are expected irrespective of habitual consumption status, whereas haemodynamic effects of caffeine may interact with this factor.

## Methods

### Initial screening

Prior to participation, volunteers were required to provide informed consent and complete an unpublished caffeine consumption questionnaire that has previously been employed to assess daily level of caffeine intake (Haskell et al. 2005). Volunteers were recruited to take part in the study if they fell into one of two pre-defined categories: ‘habitual consumers’ (those who drank tea and consumed more than 150 mg caffeine per day) or ‘non-habitual consumers’ (those who consumed less than 60 mg caffeine per day and no more than 2 cups of tea/coffee per week). These cutoffs are designed to allow the consumption of a 330 ml can of most standard colas per day in non-habitual consumers and to ensure that habitual consumers ingested the equivalent of at least three 190 ml cups of tea per day (Gray 1998). Only non-smoking volunteers who were in good health, not currently taking any dietary supplements or medication (including the contraceptive pill), were not colour-blind and did not have a history of head trauma, learning difficulties, ADHD, neurological, vascular or psychiatric illness were recruited to take part in the study.

### Design 

A double-blind, counter-balanced, within-subjects, placebo-controlled design was utilised.

### Treatment

Participants attended four study visits, at least 48 h apart and, at each, received one of the following treatments: 75 mg caffeine (pharmaceutical grade caffeine powder, Blackburn Distributions Ltd.), 50 mg L-theanine (Suntheanine, Taiyo Europe, Germany), 75 mg caffeine and 50 mg of L-theanine in combination, or placebo. The doses selected roughly equate to the levels found in two cups of tea. These were chosen in an attempt to extend previous findings exploring the effects of 100 mg L-theanine (Einother et al. 2010; Kelly et al. 2008; Owen et al. 2008) whilst more closely reflecting the ratio of L-theanine to caffeine found in tea. Each treatment was administered in the form of two capsules in order to mask any taste differences and to ensure that participants remained blind to the treatment they had received. The capsules were prepared and coded by an independent third party who had no further involvement with the study. The order in which participants received each treatment was determined by Latin square and random allocation to treatment order for each group (habitual consumers and non-habitual consumers).

### Participants

Twenty-four healthy young participants (10 males) between the ages of 18 and 35 (mean age 21.8, standard deviation (SD) 3.19) were recruited. Twelve participants were classified as habitual consumers (five males; mean age 23.3, SD 3.65) and 12 as non-habitual consumers (five males; mean age 20.4, SD 1.88). From the self-report caffeine consumption questionnaire, habitual consumers reported drinking between 163 and 432 mg caffeine per day (mean 252.2, SD 74.3). Non-habitual consumers reported drinking between 0 and 56 mg caffeine per day (mean 16.7, SD 15.6). With regards to tea consumption, habitual consumers reported consuming between 1 and 6 cups per day (mean 3.50, SD 1.46) and non-habitual consumers reported consuming between 0 and 2 per week (mean 0.45, SD 0.62). The study was approved by Northumbria University’s School of Psychology and Sport Sciences’ ethics committee and was conducted according to the Declaration of Helsinki (1964).

### Salivary caffeine levels

Saliva samples were obtained using salivettes (Sarstedt Ltd). One sample was taken upon arrival and one immediately following the post-dose cognitive assessment. This was to ensure overnight caffeine abstinence and to confirm caffeine absorption following caffeinated treatments (no analysis of post-treatment caffeine levels was made following placebo or L-theanine). Once taken, samples were frozen at −20 °C. The samples were then thawed and the caffeine levels in the saliva samples were measured using an Emit® Caffeine Assay (Dade Behring Ltd).

### Cognitive and mood assessment

All cognitive and mood measures were delivered using the Computerised Mental Performance Assessment System (COMPASS, Northumbria University, Newcastle upon Tyne, UK), a purpose-designed software application for the flexible delivery of randomly generated parallel versions of standard and novel cognitive assessment tasks. This assessment system has previously been shown to be sensitive to nutritional interventions (Kennedy et al. 2010a; Stonehouse et al. 2013) including caffeine (Kennedy and Haskell 2011). The tasks were chosen based on their known sensitivity to one or both of the nutritional interventions under investigation (Haskell et al. 2008; Kennedy and Haskell 2011; Lieberman et al. 1987) and, in order to correspond with the region of CBF measurements, their ability to activate the pre-frontal cortex (Drummond et al. 1999; Lawrence et al. 2002; Schroeter et al. 2002). The tasks completed at baseline and post-dose were identical with the exception that baseline tasks were shortened to 2 min. This ensured that participants were not connected to NIRS equipment for more than 2 h and therefore minimised the discomfort associated with restricted movement. Tasks were presented in the following order (post-dose duration in parentheses): serial 3 subtractions (4 min), serial 7 subtractions (4 min), simple reaction time (SRT) (8 min), rapid visual information processing (RVIP) (8 min), choice reaction time (CRT) (8 min) and Stroop (8 min).

### Serial 3 subtractions

A starting number between 800 and 999 appears on the screen and participants are instructed to count backwards as quickly and as accurately as possible from this number in threes, using the linear number keys to make their response. Responses are cleared when the ‘enter’ key is pressed. Participants are only shown one number on screen and the rest of the numbers are generated by subtracting from the previous number in their head. In the case of incorrect responses, subsequent responses are scored positively if they are correct in relation to the new number. This timed task was scored for total responses and number of errors.

### Serial 7 subtractions

This task is identical to the serial 3s subtraction task except that it involves the serial subtraction of 7s.

### Simple reaction time

An upwards pointing arrow appears on the screen at a random inter-stimulus duration between 1 and 3.5 s. Participants have to respond as quickly as they can when they see a stimulus appear by pressing the space bar. One hundred and ninety stimuli were presented and the task was scored for reaction time. Responses below 150 ms do not register for the task.

### Rapid visual information processing

The participant monitors a continuous series of digits for targets of three consecutive odd or three consecutive even digits. The digits are presented on the computer screen at the rate of 100 per minute in pseudo-random order, and the participant responds to the detection of a target string by pressing the space bar as quickly as possible. The task is continuous. The task was scored for percentage of target strings correctly detected, average reaction time for correct detections, and number of false alarms.

### Choice reaction time

An arrow pointing either left or right is presented in the middle of the screen at a random inter-stimulus duration between 1 to 3.5 s. As soon as participants see an arrow appear on the screen, they are required to indicate the direction of the arrow by pressing left and right keys (‘M’ and ‘Z’ on keyboard). One hundred and eighty-five stimuli were presented and the task was scored for percentage of correct responses and reaction time. Responses below 150 ms do not register.

### Stroop

In this task, a series of colour names appear on the screen one at a time in different coloured fonts. Participants are required to use a colour-coded response pad to select the colour that matches the colour font that the word is written in. The words that are presented are either ‘congruent’ (name of colour and colour of ink the same) or ‘incongruent’ (name of colour and colour of ink different) and are presented randomly. Participants are asked to respond as quickly and as accurately as possible. This timed task was scored for total responses, number of errors, reaction time and interference reaction time (difference in reaction time for congruent and incongruent stimuli). Responses below 150 ms do not register.

### Subjective assessment

Caffeine research visual analogue scales adapted from Rogers et al. (2003) that have previously been used in caffeine and L-theanine research were also included (Haskell et al. 2005, 2008; Kennedy and Haskell 2011) and were presented on-screen. Participants were shown the following descriptors ‘relaxed’, ‘alert’, ‘jittery’, ‘tired’, ‘tense’, ‘headache’, ‘overall mood’ and ‘mentally fatigued’ and asked to rate how much they matched their current state by placing an ‘x’ on a 100-mm line with the end points labelled ‘not at all’ (left hand end) and ‘extremely’ (right hand end), with the exception of ‘overall mood’, which was labelled ‘very bad’ and ‘very good’. ‘Alert’ and ‘tired’, and ‘tense’ and ‘relaxed’ scores were then combined to create respective factors of ‘alertness’ and ‘tension’ as recommended by the authors.

### Near-infrared spectroscopy measurements

Near infrared spectroscopy (NIRS) is a method of studying functional activation through monitoring changes in the haemodynamic properties of the brain (Huppert et al. 2006). It is a non-invasive brain imaging technique in which two wavelengths of light that are differentially absorbed by oxygenated and deoxygenated haemoglobin are introduced through the skull via a laser emitter and measured following transit through the upper surface of the cortex, by an optode placed at a pre-set distance from the light source.

Relative changes in the absorption of near-infrared light were measured at a time resolution of 10 Hz using a 12-channel Oxymon system (Artinis Medical Systems B.V.). The system emitted two nominal wavelengths of light (~780 and 855 nm) with an emitter/optode separation distance of 4 cm. The differential path length factor was adjusted according to the age of the participant. Relative concentration changes in oxygenated haemoglobin (oxy-Hb), deoxygenated haemoglobin (deoxy-Hb) and total haemoglobin (total-Hb) were calculated by means of a modified Beer–Lambert law using the proprietorial software. In this study, a simple two emitter/receiver optode pair configuration was utilised (i.e. two channels). The emitter/receiver optode pairs were positioned over the left and right pre-frontal cortex that included the areas corresponding to the international 10-20 system Fp1 and Fp2 EEG positions. The ability of NIRS to measure blood flow changes following cerebral activation has been validated by its use in a number of studies (Fallgatter and Strik 1998; Izzetoglu et al. 2003, 2004; Schroeter et al. 2002; Shibuya-Tayoshi et al. 2007) including following different nutritional interventions (Jackson et al. 2012a, b; Kennedy and Haskell 2011; Kennedy et al. 2010b; Wightman et al. 2014).

### Blood pressure and heart rate

Blood pressure and heart rate (Boso-Medicus Prestige; Bosch + Sohn, Germany) readings were taken from the left arm following a 5 min seated rest. Readings were taken at each visit upon arrival and following post-dose completion of the cognitive tasks.

### Procedure

**Fig. 1:**
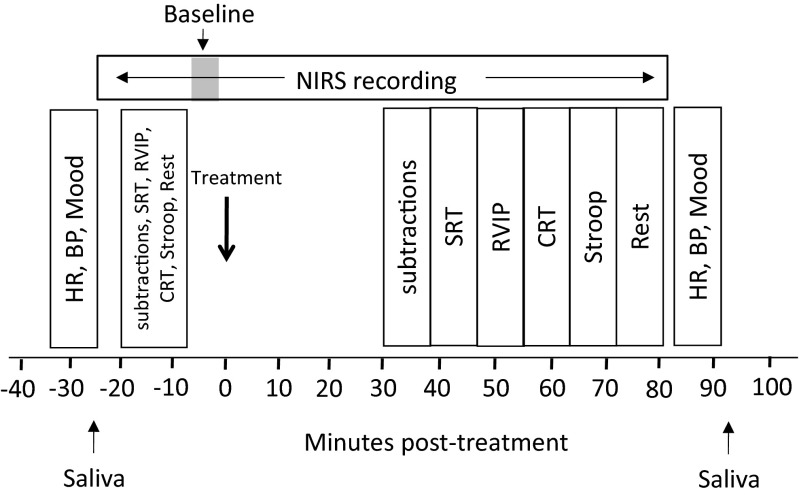
Timeline representing flow of study day

Participants were required to attend the laboratory on five separate occasions. The first visit was a screening session where participants were informed about the nature of the study, its requirements and its restrictions. Written informed consent was obtained and their eligibility to participate was confirmed. Habitual caffeine intake and source were assessed via questionnaire, and familiarisation with the tasks to be administered on the study days was conducted. The remaining four study visits were identical to each other, with the exception of the treatment administered. On each day, participants attended the lab at 8 a.m. following an overnight 12-h fast during which they were only permitted to drink water. Upon arrival, heart rate and blood pressure readings were taken following 5 min of seated rest. Following a baseline completion of the mood scales, salivary caffeine levels were taken to ensure caffeine abstinence. Following this, the NIRS headband was fitted. Participants initially sat quietly for 5 min and then made a baseline completion of the cognitive tasks. Upon completion of the tasks, participants were required to sit quietly for a 2 min NIRS resting baseline period. Participants were then required to take their treatment for the day. Following a 30 min absorption period (during which time NIRS recording continued whilst participants watched a non-stimulating wildlife DVD), participants completed a second set of the cognitive tasks and a final rest period (8 min) (included to allow an assessment of whether any CBF effects apparent after the absorption period are as a result of increased neural demand during tasks or are simply due to the time course of effects of treatment). They then had their blood pressure and heart rate measured for a second time, rated their mood for a second time, and gave a second saliva sample, used to confirm caffeine absorption following caffeinated treatment (see Fig. 1 for more details of procedure and task duration). Participants returned for their next study visit within 7 days, following (at least) a 48-h washout period.

### Statistics

To assess the possibility of any on-day or consumer status differences in cognitive performance, mood, blood pressure and heart rate prior to treatment, two-way repeated measures ANOVAs were conducted (treatment × consumer status) on baseline data. Any significant differences were further explored with Bonferroni-corrected pairwise comparisons.

In order to confirm caffeine absorption for caffeine-containing treatments (caffeine, combination), a two-way mixed ANOVA was conducted (treatment × time) on pre- and post-caffeine and combination saliva samples.

NIRS data was averaged across 4 min (absorption period) and 8 min (individual tasks) epochs and baseline adjusted to the 2 min post-task resting pre-treatment period. To account for disruption in NIRS readings as a result of consumption of treatment, the first 2 min of the absorption period was omitted from the analysis. Data from both channels were averaged for oxy-Hb and deoxy-Hb and analysed by three-way mixed ANOVA (epoch × treatment × consumer status). Significant treatment-related interactions were further investigated by a priori planned comparisons where each active treatment was compared to placebo at each epoch utilising *t* tests calculated with the mean squares error from the ANOVA (Keppel 1991). In order to reduce the potential for type I errors, only those planned comparisons associated with a statistically significant difference on the initial ANOVA are reported.

Cognitive performance, subjective mood, heart rate and blood pressure data were analysed using the MIXED procedure in SPSS 21.0. The model included the respective baseline as a time variant covariate, and fixed effects terms entered into the model were treatment, consumer, treatment*consumer and baseline value. Significant effects or interactions (*p* < 0.05) were further explored with Bonferroni-corrected pairwise comparisons.

## Results

### Baseline measures

A significant on-day pre-treatment difference in baseline Stroop errors [F(3, 66) = 3.13, *p* < 0.05] was lost following Bonferroni correction. There were no other significant on-day or consumer status differences in cognitive performance, mood or autonomic measures prior to treatment.

### Salivary caffeine levels

All participants adhered to overnight caffeine restriction. Mean baseline values were 0.34 μg/ml and confirmed overnight abstinence [levels below 1 μg/ml have previously been reported for overnight caffeine abstinence (Evans and Griffiths 1999)]. Following caffeinated treatment, salivary caffeine levels were 2.25 μg/ml (combination) and 2.33 μg/ml (caffeine). Analysis of the results confirmed that in comparison to baseline levels, salivary caffeine was significantly higher following caffeine and combination treatments [F(1, 23) = 63.27, *p* < 0.0001], with no significant difference between treatments.

### Central haemodynamic effects (NIRS)

#### Oxygenated haemoglobin

**Fig. 2:**
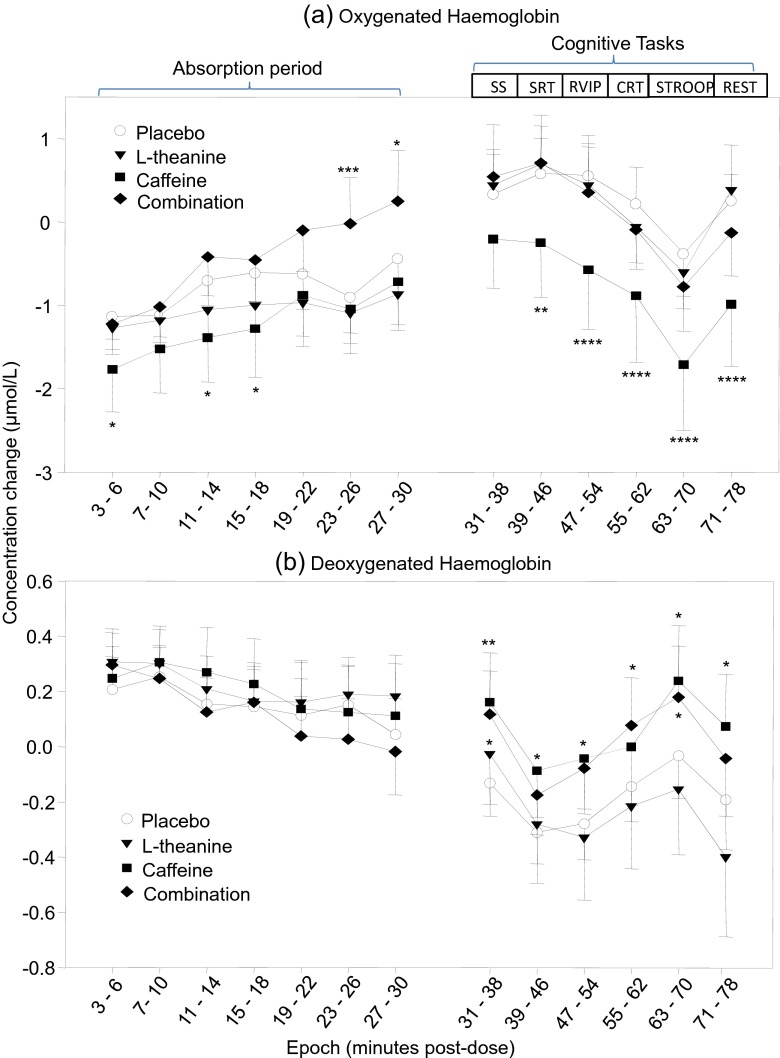
Mean change in concentration of NIRS parameters (oxy-Hb and deoxy-Hb) during absorption period and cognitive tasks following placebo (*circle*), 50 mg L-theanine (*inverted triangle*), 75 mg caffeine (*square*) and a combination of 50 mg L-theanine and 75 mg caffeine (*diamond*). Treatment × epoch interaction effects are shown for **a** oxygenated haemoglobin and **b** deoxygenated haemoglobin (*p < 0.05; ** *p* < 0.01; *** *p* < 0.005; **** *p* < 0.001)

A significant interaction effect (epoch × treatment) was observed for oxy-Hb [*F*(36, 792) = 1.503, *p* < 0.05]. Oxy-Hb was significantly reduced during minutes 3–6 (*p* < 0.05) and 11–18 of the absorption period (*p* < 0.05) and during SRT (*p* < 0.01), RVIP (*p* < 0.001), CRT (*p* < 0.001) and Stroop tasks (*p* < 0.001) and the rest period (*p* < 0.001) following caffeine as compared to placebo. This effect was not evident during cognitive tasks when caffeine was combined with L-theanine, or when L-theanine was administered alone. Following the combination, oxy-Hb was significantly increased during minutes 23–30 of the absorption period as compared to placebo [minutes 23–26 (*p* < 0.005) and 27–30 (*p* < 0.05)]; see Fig. 2a.

#### Deoxygenated haemoglobin

A significant interaction effect (epoch × treatment) was also evinced for deoxy-Hb [*F*(36, 792) = 1.617, *p* < 0.05]. Significant increases were observed during serial subtractions (*p* < 0.01), SRT (*p* < 0.05), RVIP (*p* < 0.05) and Stroop tasks (*p* < 0.05) and during the rest period (*p* < 0.05) following caffeine as compared to placebo. Following the combination, deoxy-Hb was significantly increased during serial subtractions (*p* < 0.05), CRT (*p* < 0.05) and Stroop tasks (*p* < 0.05), as compared to placebo; see Fig. 2b.

**Fig. 3:**
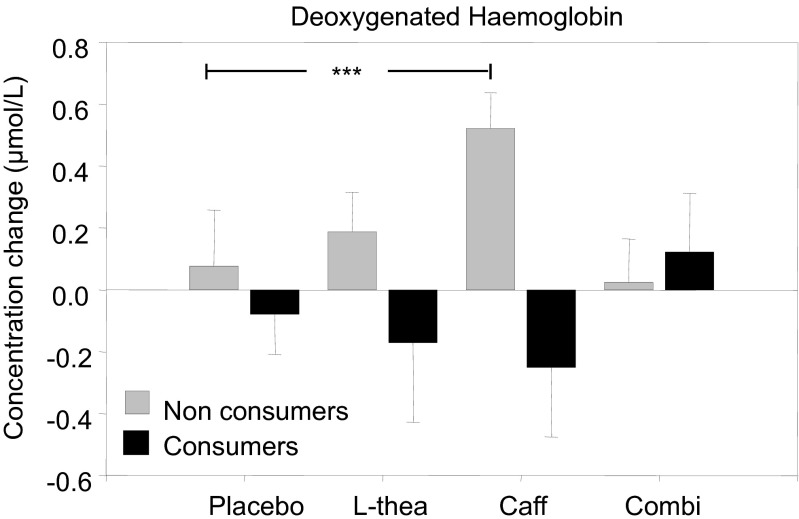
Mean change in concentration of deoxy-Hb overall following placebo, 50 mg L-theanine (*L-thea*), 75 mg caffeine (*Caff*) and a combination of 50 mg L-theanine and 75 mg caffeine (*Combi*) per consumer status. Treatment × consumer status interaction effects are shown for deoxygenated haemoglobin (***p < 0.005)

There was also a significant treatment by consumer status interaction for deoxy-Hb [*F*(3, 792) = 3.250, *p* < 0.05]. Non-habitual consumers had significantly higher deoxy-Hb throughout the absorption and task periods following caffeine as compared to placebo (*p* < 0.005); see Fig 3.

### Behavioural effects

#### Serial 3 subtractions

A significant treatment × consumer status interaction effect on the total number of serial three subtractions [F(3, 66) = 3.40, *p* < 0.05] was evinced. However, pairwise comparisons revealed no significant differences between treatments.

#### Choice reaction time

There was a significant main effect of treatment on choice reaction time [*F*(3, 66) = 4.18, *p* < 0.05]. Reaction time was significantly faster following caffeine compared to placebo (*p* < 0.05). There was also a significant consumer status effect [*F*(1, 66) = 10.08, *p* < 0.005] whereby consumers were found to be significantly faster than non-habitual consumers, irrespective of treatment.

#### Stroop

There was a significant main effect of treatment on total number of Stroop responses [*F*(3, 66) = 8.95, *p* < 0.0001]. The number of responses was significantly higher following caffeine compared to placebo (*p* < 0.005). There was also a significant main effect of treatment on Stroop errors [*F*(3, 66) = 3.18, *p* < 0.05]. Participants produced less errors following L-theanine as compared to the combination (*p* < 0.05). A main effect of treatment on Stroop reaction time [F(3, 66) = 6.92, *p* < 0.005] demonstrated significantly faster responses following caffeine compared to placebo (*p* < 0.005). There was also a significant consumer status effect on Stroop reaction time interference [F(1, 66) = 5.16, *p* < 0.05], whereby habitual consumers showed a greater interference effect than habitual non-consumers. Unadjusted means and standard error for cognitive parameters can be found in Table 1.

**Table 1:** Unadjusted baseline and post-dose scores for cognitive tasks for each treatment by consumer status

Measure	Treatment	*n*	Pre-dose baseline score		Post-dose score		Treatment effect	Consumer effect	Treatment × consumer interaction
			Consumers	Non-consumers	Consumers	Non-consumers			
**Serial 3s total responses (number)**	Placebo	12/12	40.17 ± 4.53	41.08 ± 3.59	79.75 ± 8.70	81.67 ± 7.24	F = 1.48 *p* > 0.1	F < 1	**F** = **3.40** ***p*** = **0.04**
	L-theanine		40.58 ± 4.07	42.92 ± 4.36	84.83 ± 7.47	79.50 ± 8.19			
	Caffeine		42.25 ± 3.25	43.67 ± 5.26	82.33 ± 6.69	85.25 ± 8.05			
	Combination		40.00 ± 3.24	40.75 ± 3.95	84.58 ± 6.63	83.33 ± 7.17			
Serial 3s errors (number)	Placebo	12/12	1.50 ± 0.42	1.25 ± 0.54	3.92 ± 0.85	3.25 ± 0.79	F = 1.12 *p* > 0.1	F < 1	F < 1
	L-theanine		1.58 ± 0.50	1.42 ± 0.51	2.75 ± 0.59	4.08 ± 0.76			
	Caffeine		2.50 ± 0.65	1.58 ± 0.40	3.58 ± 0.67	3.08 ± 0.60			
	Combination		2.58 ± 0.51	1.25 ± 0.30	2.67 ± 0.43	2.58 ± 0.54			
Serial 7s total responses (number)	Placebo	12/12	25.08 ± 2.97	25.83 ± 3.47	53.75 ± 5.23	54.08 ± 5.28	F = 1.22 *p* > 0.1	F = 1.17 *p* > 0.1	F < 1
	L-theanine		27.00 ± 2.20	24.83 ± 2.86	54.08 ± 4.66	49.33 ± 5.05			
	Caffeine		25.58 ± 2.29	26.08 ± 2.79	53.83 ± 4.38	51.33 ± 5.71			
	Combination		26.33 ± 2.05	24.75 ± 2.37	56.42 ± 4.43	51.08 ± 4.41			
Serial 7s errors (number)	Placebo	12/12	1.50 ± 0.40	1.25 ± 0.45	3.67 ± 0.89	3.50 ± 1.15	F < 1	F < 1	F < 1
	L-theanine		1.33 ± 0.36	2.50 ± 0.76	2.58 ± 0.53	3.75 ± 1.21			
	Caffeine		1.67 ± 0.68	2.00 ± 0.54	3.25 ± 0.74	4.00 ± 0.72			
	Combination		1.58 ± 0.45	2.08 ± 0.70	3.33 ± 0.61	3.58 ± 0.73			
SRT (ms)	Placebo	12/12	294.00 ± 11.19	326.54 ± 16.59	308.38 ± 11.85	344.09 ± 15.26	F = 2.63 *p* = 0.08	F = 2.06 *p* > 0.1	F < 1
	L-theanine		288.71 ± 11.38	329.85 ± 19.73	308.37 ± 11.77	365.31 ± 20.98			
	Caffeine		304.90 ± 9.96	308.08 ± 9.96	312.21 ± 13.43	320.93 ± 11.26			
	Combination		290.87 ± 9.64	322.86 ± 24.60	304.60 ± 11.62	340.73 ± 28.58			
RVIP accuracy (%)	Placebo	12/11	70.83 ± 6.17	62.50 ± 4.30	59.51 ± 6.61	52.70 ± 3.41	F = 1.09 *p* > 0.1	F < 1	F = 1.52 *p* > 0.1
	L-theanine		68.23 ± 5.63	67.05 ± 4.85	58.33 ± 5.20	56.68 ± 3.80			
	Caffeine		75.52 ± 7.19	62.50 ± 6.31	62.24 ± 6.61	60.94 ± 4.67			
	Combination		68.75 ± 8.43	67.05 ± 5.34	64.19 ± 5.87	60.23 ± 5.10			
RVIP RT (ms)	Placebo	12/11	502.55 ± 18.02	481.36 ± 15.76	513.36 ± 21.03	482.54 ± 11.00	F = 1.79 *p* > 0.1	F = 2.99 *p* > 0.1	F < 1
	L-theanine		501.75 ± 18.82	480.70 ± 13.99	511.05 ± 17.77	471.87 ± 13.28			
	Caffeine		510.43 ± 18.23	487.52 ± 12.51	512.74 ± 18.63	474.19 ± 10.02			
	Combination		499.29 ± 27.41	471.37 ± 14.31	498.83 ± 17.93	465.15 ± 14.47			
RVIP false alarms (number)	Placebo	12/11	1.58 ± 0.50	2.18 ± 0.52	4.42 ± 0.91	5.45 ± 1.23	F < 1	F = 1.12 *p* > 0.1	F < 1
	L-theanine		1.17 ± 0.30	1.73 ± 0.49	3.92 ± 1.18	7.09 ± 2.50			
	Caffeine		0.83 ± 0.24	1.91 ± 0.49	3.42 ± 0.75	5.36 ± 2.64			
	Combination		0.83 ± 0.30	1.27 ± 0.38	3.83 ± 0.90	6.45 ± 1.92			
CRT accuracy (%)	Placebo	12/12	96.17 ± 0.87	96.67 ± 0.79	96.04 ± 0.98	96.08 ± 1.16	F = 1.19 *p* > 0.1	F < 1	F < 1
	L-theanine		96.33 ± 0.69	95.33 ± 1.68	96.31 ± 0.88	96.22 ± 0.81			
	Caffeine		96.83 ± 0.72	94.50 ± 0.82	96.40 ± 1.03	96.26 ± 0.73			
	Combination		96.50 ± 0.96	95.17 ± 1.19	96.71 ± 0.75	96.40 ± 0.66			
**CRT (ms)**	Placebo	12/12	425.90 ± 15.09	437.36 ± 18.00	423.10 ± 15.16	496.86 ± 27.84	**F** = **4.18** ***p*** = **0.02**	**F** = **10.08** ***p*** = **0.004**	F = 2.68 *p* = 0.07
	L-theanine		420.64 ± 10.14	448.00 ± 24.75	420.44 ± 9.84	480.83 ± 25.21			
	Caffeine		410.14 ± 10.90	453.22 ± 27.21	406.85 ± 11.27	442.59 ± 17.51			
	Combination		409.81 ± 10.06	428.49 ± 25.41	406.70 ± 9.31	448.55 ± 20.78			
**Stroop total responses (number)**	Placebo	12/12	73.33 ± 1.16	74.42 ± 1.13	294.00 ± 4.03	290.75 ± 4.67	**F** = **8.95** ***p*** **< 0.001**	F < 1	F < 1
	L-theanine		73.42 ± 1.94	72.08 ± 1.97	295.42 ± 4.53	289.33 ± 6.16			
	Caffeine		73.92 ± 1.00	73.92 ± 1.73	300.17 ± 3.91	299.33 ± 4.59			
	Combination		74.83 ± 1.06	73.42 ± 1.28	300.00 ± 3.14	297.25 ± 3.27			
**Stroop errors (number)**	Placebo	12/12	1.33 ± 0.36	2.00 ± 0.51	9.58 ± 3.10	11.00 ± 2.05	**F** = **3.18** ***p*** = **0.04**	F < 1	F < 1
	L-theanine		20.8 ± 0.56	2.33 ± 0.43	8.92 ± 2.62	8.25 ± 1.82			
	Caffeine		2.50 ± 0.82	3.08 ± 0.79	9.25 ± 3.04	10.83 ± 2.26			
	Combination		2.00 ± 0.81	1.92 ± 0.62	12.00 ± 4.34	10.42 ± 1.93			
**Stroop RT (ms)**	Placebo	12/12	651.67 ± 26.36	628.92 ± 26.50	641.25 ± 22.65	660.33 ± 28.40	**F** = **6.92** ***p*** = **0.002**	F < 1	F < 1
	L-theanine		659.50 ± 49.91	692.92 ± 57.09	637.08 ± 25.40	672.17 ± 40.69			
	Caffeine		633.67 ± 23.40	643.17 ± 40.67	607.83 ± 21.39	613.08 ± 26.12			
	Combination		615.67 ± 22.50	651.33 ± 31.57	605.67 ± 17.21	622.08 ± 18.30			
**Stroop interference RT (ms)**	Placebo	12/12	11.33 ± 15.55	12.67 ± 12.96	32.92 ± 7.86	7.92 ± 8.60	F = 1.23 *p* > 0.1	**F** = **5.16** ***p*** = **0.03**	F = 2.13 *p* > 0.1
	L-theanine		25.00 ± 15.19	14.17 ± 14.36	43.92 ± 9.26	17.67 ± 4.46			
	Caffeine		38.67 ± 14.67	28.75 ± 19.31	29.08 ± 9.32	29.00 ± 6.65			
	Combination		25.08 ± 20.05	28.83 ± 10.99	45.33 ± 7.76	27.08 ± 12.09			

Means ± SEM values are presented with F and *p* values from the primary analysis of main effects and treatment × consumer interactions. Significant measures are shown in bold

*SRT* simple reaction time, *RVIP* rapid visual information processing, *CRT* choice reaction time

#### Alert

There was a significant main effect of treatment on alert ratings [*F*(3, 66) = 3.09, *p* < 0.05]. Pairwise comparisons revealed no significant differences following Bonferroni correction.

#### Overall mood

There was a significant main effect of treatment on ratings of mood [*F*(3, 66) = 3.13, *p* < 0.05]. Overall mood ratings were significantly higher following caffeine as compared to placebo (*p* < 0.005). Unadjusted means and standard error for mood parameters can be found in Table 2.

**Table 2:** Unadjusted baseline and post-dose ratings for mood for each treatment by consumer status

Measure	Treatment	*n*	Pre-dose baseline score		Post-dose score		Treatment effect	Consumer effect	Treatment × consumer interaction
			Consumers	Non-consumers	Consumers	Non-consumers			
Relaxed (mm)	Placebo	12/12	64.75 ± 2.73	63.83 ± 3.64	51.83 ± 5.09	55.33 ± 5.47	F = 2.28 *p* > 0.1	F < 1	F < 1
	L-theanine		55.83 ± 5.76	63.50 ± 4.76	56.00 ± 5.57	62.58 ± 4.80			
	Caffeine		58.83 ± 6.63	62.25 ± 6.10	51.50 ± 5.59	54.42 ± 7.35			
	Combination		61.17 ± 4.24	67.92 ± 4.64	54.75 ± 6.32	64.42 ± 4.91			
**Alert (mm)**	Placebo	12/12	57.17 ± 5.63	50.17 ± 6.07	51.83 ± 3.04	51.50 ± 6.00	**F** = **3.09** ***p*** = **0.048**	F < 1	F < 1
	L-theanine		51.50 ± 4.09	58.42 ± 6.34	47.92 ± 6.24	54.75 ± 6.75			
	Caffeine		55.83 ± 4.65	49.17 ± 6.98	58.42 ± 4.73	57.92 ± 6.80			
	Combination		59.25 ± 3.12	54.75 ± 6.30	56.92 ± 4.95	57.75 ± 5.73			
Jittery (mm)	Placebo	12/12	28.92 ± 5.51	17.75 ± 3.86	37.75 ± 6.78	23.00 ± 5.40	F < 1	F = 1.48 *p* > 0.1	F < 1
	L-theanine		26.92 ± 3.59	24.25 ± 6.27	36.08 ± 6.36	25.92 ± 6.59			
	Caffeine		33.33 ± 4.32	27.17 ± 5.33	36.25 ± 5.93	30.92 ± 6.88			
	Combination		26.50 ± 5.26	26.58 ± 6.40	35.67 ± 6.23	25.08 ± 5.84			
Tired (mm)	Placebo	12/12	46.25 ± 7.87	54.25 ± 6.70	51.67 ± 6.59	53.33 ± 5.83	F = 2.68 *p* = 0.07	F < 1	F < 1
	L-theanine		45.75 ± 6.44	42.83 ± 4.36	45.67 ± 6.91	49.25 ± 6.36			
	Caffeine		50.92 ± 5.83	53.92 ± 6.41	40.25 ± 4.84	44.67 ± 7.95			
	Combination		39.08 ± 6.36	49.67 ± 5.88	46.83 ± 6.24	43.83 ± 7.08			
Tense (mm)	Placebo	12/12	28.50 ± 4.27	24.42 ± 4.89	36.17 ± 6.30	23.42 ± 4.78	F < 1	F = 1.51 *p* > 0.1	F = 1.06 *p* > 0.1
	L-theanine		34.00 ± 5.56	25.83 ± 5.35	38.83 ± 6.36	22.92 ± 5.65			
	Caffeine		32.17 ± 4.50	26.08 ± 6.01	35.08 ± 6.14	29.58 ± 6.71			
	Combination		31.75 ± 6.20	18.67 ± 3.28	32.92 ± 5.72	25.58 ± 5.96			
Headache (mm)	Placebo	12/12	16.50 ± 6.34	18.00 ± 5.39	28.58 ± 7.11	36.83 ± 7.40	F = 1.27 *p* > 0.1	F < 1	F = 1.12 *p* > 0.1
	L-theanine		16.00 ± 4.57	20.50 ± 5.93	28.42 ± 6.36	30.83 ± 6.50			
	Caffeine		19.83 ± 6.58	14.83 ± 3.79	31.25 ± 7.62	27.92 ± 6.85			
	Combination		12.75 ± 3.59	17.92 ± 4.80	26.25 ± 6.43	21.00 ± 5.55			
**Overall mood (mm)**	Placebo	12/12	67.33 ± 4.43	59.83 ± 4.03	59.33 ± 3.70	55.92 ± 5.14	**F** = **3.13** ***p*** = **0.046**	F < 1	F = 1.38 *p* > 0.1
	L-theanine		58.50 ± 4.04	66.08 ± 4.08	58.33 ± 4.20	63.67 ± 4.44			
	Caffeine		61.50 ± 3.98	59.25 ± 4.34	62.00 ± 3.91	64.83 ± 4.92			
	Combination		66.33 ± 4.02	66.50 ± 3.97	61.17 ± 4.13	68.42 ± 4.02			
Mental fatigue (mm)	Placebo	12/12	35.83 ± 7.27	30.50 ± 5.59	52.75 ± 7.21	48.25 ± 5.33	F = 2.61 *p* = 0.08	F < 1	F < 1
	L-theanine		39.25 ± 5.38	35.00 ± 5.00	54.00 ± 5.36	46.17 ± 5.65			
	Caffeine		29.25 ± 5.20	33.83 ± 6.34	44.42 ± 7.01	45.17 ± 7.33			
	Combination		30.58 ± 6.34	31.67 ± 6.13	45.17 ± 6.55	37.33 ± 5.47			

Means ± SEM values are presented with F and *p* values from the primary analysis of main effects and treatment × consumer interactions. Significant measures are shown in bold

### Peripheral haemodynamic effects

#### Blood pressure and heart rate

Analysis of blood pressure levels revealed a significant main effect of treatment for systolic [*F*(3, 66) = 5.50, *p* < 0.01] and diastolic [*F*(3, 66) = 3.37, *p* < 0.05] blood pressure. Following the combination treatment, systolic blood pressure was significantly higher as compared to placebo (*p* < 0.05) and L-theanine (*p* < 0.05). Diastolic blood pressure was also significantly higher following the combination in comparison to placebo (*p* < 0.01). There were no significant effects of treatment on heart rate. Unadjusted means and standard error for peripheral haemodynamic parameters can be found in Table 3.

**Table 3:** Unadjusted baseline and post-dose blood pressure and heart rate readings for each treatment by consumer status

Measure	Treatment	*n*	Pre-dose baseline score		Post-dose score		Treatment effect	Consumer effect	Treatment × consumer interaction
			Consumers	Non-consumers	Consumers	Non-consumers			
**Systolic blood pressure (mmHg)**	Placebo	12/12	115.75 ± 3.70	111.83 ± 2.87	114.92 ± 3.47	114.50 ± 1.67	**F** = **5.50** ***p*** = **0.006**	F = 1.69 *p* > 0.1	F = 2.90 *p* = 0.06
	L-theanine		120.58 ± 5.07	114.58 ± 3.58	119.67 ± 3.69	111.17 ± 2.94			
	Caffeine		115.42 ± 2.90	111.08 ± 2.39	119.67 ± 3.88	114.75 ± 2.47			
	Combination		116.92 ± 3.30	113.33 ± 3.35	124.50 ± 4.07	117.00 ± 2.48			
**Diastolic Blood pressure (mmHg)**	Placebo	12/12	76.75 ± 2.38	75.33 ± 1.51	75.75 ± 3.95	75.75 ± 2.28	**F** = **3.73** ***p*** = **0.03**	F = 1.38 *p* > 0.1	F = 1.17 *p* > 0.1
	L-theanine		78.17 ± 2.00	73.25 ± 1.72	82.25 ± 2.47	76.58 ± 1.75			
	Caffeine		75.00 ± 1.62	72.33 ± 1.58	83.08 ± 2.67	75.00 ± 2.56			
	Combination		76.75 ± 2.23	72.92 ± 1.64	81.83 ± 2.88	77.33 ± 1.83			
Heart rate (bpm)	Placebo	12/12	68.92 ± 2.99	70.33 ± 2.78	62.08 ± 2.65	62.17 ± 2.33	F < 1	F < 1	F < 1
	L-theanine		67.92 ± 3.53	70.83 ± 2.74	61.50 ± 2.01	65.00 ± 2.14			
	Caffeine		65.00 ± 2.54	67.58 ± 2.90	59.25 ± 2.68	63.25 ± 3.59			
	Combination		68.17 ± 2.89	71.42 ± 2.10	62.17 ± 3.00	63.25 ± 2.05			

Means ± SEM values are presented with F and *p* values from the primary analysis of main effects and treatment × consumer interactions. Significant measures are shown in bold

## Discussion

The primary aim of the current study was to assess, for the first time, the independent and interactive effects of caffeine and L-theanine on cerebral blood flow. The study also extended previous cognitive and mood findings of the two substances by exploring behavioural effects of doses lower than those used in previous studies of a similar methodology, which more closely reflect the ratios present in tea. The results here demonstrate that, compared to placebo, caffeine led to a reduction in oxygenated haemoglobin in the pre-frontal cortex during minutes 3–6 and 11–18 of the absorption period and during task performance commencing at 39 min post-dose until the end of testing. This effect of caffeine was abolished by combining it with L-theanine, despite no effects of L-theanine in isolation on this measure. The expected reduction in deoxygenated haemoglobin during the task period was attenuated following both of the treatments that contained caffeine, as compared to placebo. Following caffeine alone, this effect reached significance during the entire task period with the exception of CRT, whereas following the combination, this effect reached significance only during serial subtractions, CRT and Stroop. In addition, there was a significant treatment × consumer interaction, whereby the effects on deoxygenated haemoglobin following caffeine alone were predicated on an increase in non-habitual consumers throughout the entire testing session.

The reduction in oxy-Hb in the current study supports findings from previous imaging studies showing decreased CBF following higher doses of caffeine (Chen and Parrish 2009a; Rack-Gomer et al. 2009), including in the form of tea (Vidyasagar et al. 2013), and a reduction in total haemoglobin observed in a more recent study utilising NIRS that administered the same, comparatively low, dose of caffeine as here (Kennedy and Haskell 2011). Of particular interest, the effects of caffeine on oxy-Hb, but not deoxy-Hb, were abolished by co-administration with L-theanine. This pattern of effects is consistent with those previously observed on blood pressure by Rogers et al. (2008), showing attenuation of the rise in blood pressure following caffeine in isolation when it was combined with L-theanine. Although higher doses of caffeine and L-theanine were used in that study (250 and 200 mg respectively), the ratio of compounds was more comparable to those used here than other studies in this area, and the post-treatment assessment took place within a similar time frame (45 min post-dose).

The significant increase in deoxy-Hb observed following caffeine relative to placebo is contrary to the decrease in deoxy-Hb generally observed during cognitive demand (Fallgatter and Strik 1998; Izzetoglu et al. 2003, 2004; Kennedy et al. 2010b; Shibuya-Tayoshi et al. 2007). It is, however, not unexpected given previously observed neurovascular uncoupling following caffeine (Laurienti et al. 2003; Mulderink et al. 2002; Perthen et al. 2008). Data from calibrated (to hypercapnia) BOLD fMRI studies showing that a high single dose of caffeine (~200 mg+), administered to habitual consumers, reduces blood flow but increases oxygen consumption in response to task/stimulation, suggest that this increase in deoxy-Hb is indicative of neural activation (Chen and Parrish 2009b; Perthen et al. 2008); this is supported by a lack of effects on deoxy-Hb during the absorption period. Laurienti et al. (2002) also showed that the effects of 250 mg caffeine administration on fMRI BOLD were correlated to dietary caffeine use, with high consumers showing significantly higher signal change than low consumers. A differential effect of consumer status on deoxy-Hb in the current study, reflecting a significant increase across tasks following caffeine in non-habitual consumers only, suggests that the disparity of effects of caffeine on BOLD is the result of varying deoxy-Hb rather than oxy-Hb response. Given the lack of interaction effect on oxy-Hb in the current study, this may point to a specific tolerance to the effects on deoxy-Hb in habitual caffeine consumers. As the effects on deoxy-Hb suggest increased neural activation in non-consumers, this would indicate an upregulation of A_1_ receptors (Laurienti et al. 2003), as has previously been demonstrated (Johansson et al. 1993).

In relation to the behavioural effects, caffeine significantly reduced choice reaction time, improved Stroop performance and improved subjective ratings of overall mood. The tasks affected by caffeine were the latter two completed in the cognitive paradigm, potentially indicating that effects on behaviour only became apparent 55 min post-administration. However, it is interesting to note that these tasks also elicited the smallest rise in oxy-Hb in all conditions, presumably as a result of decreased activation over time. Despite effects of caffeine on oxy-Hb and/or deoxy-Hb during serial subtractions, simple reaction time and RVIP, there were no corresponding behavioural effects on these tasks. The only other behavioural effect of treatment was manifested as significantly less Stroop errors following L-theanine in isolation compared to the combination treatment. There were no positive effects on behaviour of combining L-theanine and caffeine. This is contrary to previous findings (Einother et al. 2010; Haskell et al. 2008; Kelly et al. 2008; Owen et al. 2008) and is also somewhat surprising given that the combination showed a similar but lesser modulation of deoxy-Hb to caffeine, but did not decrease oxy-Hb (in fact, this was increased during the final two epochs of the absorption period). The lack of behavioural effects may be due to the lower doses used here and the ratio of compounds favouring caffeine as opposed to L-theanine (see Camfield et al. 2014 for review of behavioural effects of tea constituents). An overall consumer status effect indicated that habitual consumers were significantly faster on the CRT task as compared to non-habitual consumers, irrespective of treatment. Non-habitual consumers showed less reaction time interference on the Stroop task than habitual consumers. However, examination of means revealed that habitual consumers were faster than non-habitual consumers in response to both congruent and incongruent stimuli. The lack of interactive effects between treatment and consumer status would seem to indicate net effects of caffeine (Haskell et al. 2005; Hewlett and Smith 2006) rather than reversal of withdrawal (Rogers et al. 2003). However, it is important to note that only five of the non-habitual consumers in the current study reported not consuming any caffeine, and therefore, the potential impact of caffeine withdrawal in this group, consuming an average of 16.7 mg caffeine per day, cannot be categorically ruled out (James 2014).

In terms of the peripheral effects, the combination treatment was found to increase systolic blood pressure compared to L-theanine and placebo and increased diastolic blood pressure compared to placebo. The ability of caffeine to raise blood pressure has been documented previously (James 2004; Rogers et al. 2008; Umemura et al. 2006); therefore, the rise seen in the present study observed following caffeine combined with L-theanine, but not caffeine in isolation, is unexpected. This finding is also in contrast to the findings of Rogers et al. (2008), who found that when 250 mg caffeine was combined with 200 mg L-theanine, it was able to attenuate the rise in blood pressure seen following caffeine in isolation. However, Giesbrecht et al. (2010) found that 40 mg caffeine combined with 97 mg L-theanine led to significant increases in systolic blood pressure with a trend towards the same for diastolic. These contrasting effects of caffeine/L-theanine combinations on blood pressure may be indicative of differential effects of different doses and ratios on this parameter.

It is now largely accepted that, at normal dietary levels, the mechanism of action of caffeine is through antagonism of adenosine A_1_ and A_2A_ receptors (Fredholm et al. 1999), increasing release of excitatory neurotransmitters and neuronal firing rate at A_1_ receptors (Koppelstaetter et al. 2008) and decreasing blood flow at A_2A_ receptors (Laurienti et al. 2003). Turning to L-theanine, since the majority of research into the neuropharmacology of L-theanine has been conducted in animals, the mechanism by which it modulates physiological and behavioural measures in humans is less well established, although there is evidence to suggest that L-theanine activates the ERK/eNOS pathway in vitro, thereby increasing endothelial nitric oxide production, a key mediator in vascular function (Siamwala et al. 2013), a finding which may play a role in L-theanine’s previously observed effects on blood pressure (Rogers et al. 2008). Animal studies have demonstrated that it can dose-dependently increase levels of dopamine in the rat striatum (Yokogoshi et al. 1998), increase gamma-aminobutyric acid (GABA) following intraperitoneal administration to mice (Kimura and Murata 1971) and modulate serotonin levels (Yokogoshi et al. 1995, 1998). L-theanine has also been shown to antagonise glutamate receptors (Kakuda et al. 2002), a finding which has been linked to neuroprotection against cerebral ischemia in mice and gerbils (Egashira et al. 2004; Kakuda et al. 2000). Given the propensity for consumption of caffeine with L-theanine, further work is warranted exploring the potential modulatory mechanisms of L-theanine.

The present study has demonstrated that caffeine and L-theanine, at doses equivalent to one to two cups of tea, are capable of modulating cerebral haemodynamics, cognitive performance, mood and autonomic measures. When combined with 75 mg caffeine, 50 mg L-theanine abolished a reduction in oxy-Hb observed following 75 mg caffeine in isolation, with this effect still apparent at the end of the assessment period. Although post-dose testing took place during peak times for caffeine and L-theanine, ranging from 15 to 120 min (Fredholm et al. 1999) and 32 to 55 min (van der Pijl et al. 2010) respectively, future studies should aim to determine the duration of L-theanine’s effects on caffeine, by extending the post-dose testing period. This study also demonstrated increases in deoxy-Hb during cognitive tasks, with effects of caffeine on this measure being exaggerated in habitual non-consumers of caffeine, providing partial replication of a previous study showing modulation of the CBF effects of 75 mg caffeine as a function of habitual caffeine consumption (Kennedy and Haskell 2011). In the current study, improvements to behaviour observed following 75 mg caffeine in isolation, irrespective of consumer status, were lost when combined with L-theanine. Therefore, the results presented here do not support combining caffeine with L-theanine at the doses tested, but it should be noted that when consumed in the form of tea, there are many other compounds present with the potential to interact with the two compounds explored here, and further exploration of these interactions is warranted. Given the evidence for differential dose effects of the compounds studied, in terms of behaviour and haemodynamics, it is also vital that future research explores the effects of lower doses, equivalent to one cup of tea.
